# Supplementary material for: Changes in ImPACT Cognitive Subtest Networks Following Sport-Related Concussion
Source: Brain Sci. 2023 Jan 20;13(2):177. doi: 10.3390/brainsci13020177 (PMC9953817; doi:10.3390/brainsci13020177)
Supplement: Supplementary file 1 [file brainsci-13-00177-s001.zip › brainsci-2142716-supplementary.pdf]

Supplementary Material

Figure S1. Diagram of Participant Selection.

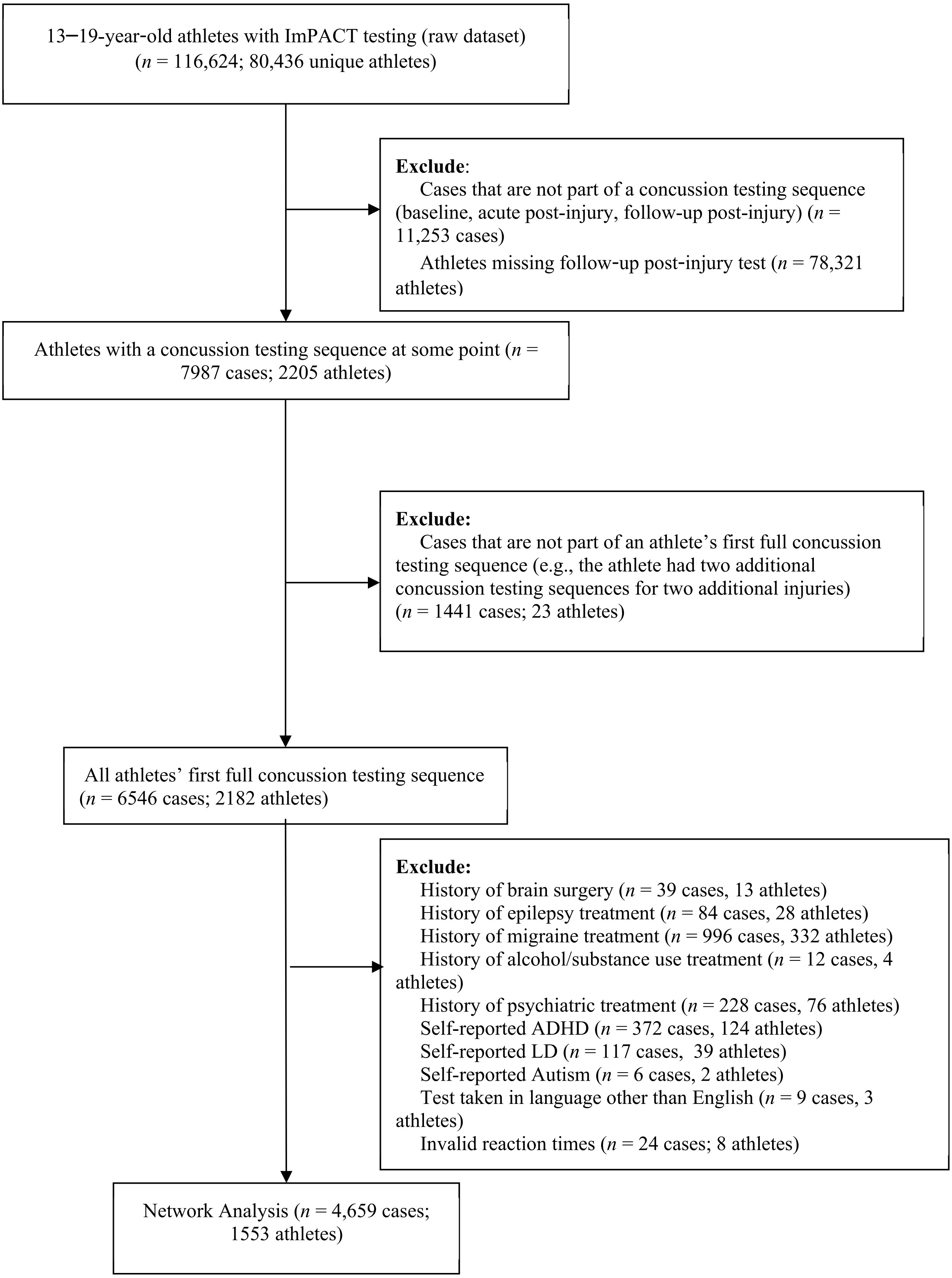

Table S1. Means and Standard Deviations of Cognitive Variables.

**Cognitive Variable**	**T1**	**T2**	**T3**
	Mean (SD)	Mean (SD)	Mean (SD)
Word Memory Hits	11.58 (0.74)	10.66 (1.68)	11.17 (1.19)
Word Memory Correct Distractors	11.63 (0.79)	10.53 (2.02)	11.40 (1.12)
Word Memory Hits (delay)	10.53 (1.39)	9.49 (2.06)	9.79 (1.95)
Word Memory Correct Distractors (delay)	10.81 (1.57)	9.56 (2.36)	10.59 (1.71)
Design Memory Hits	10.08 (1.45)	8.97 (1.84)	9.71 (1.79)
Design Memory Correct Distractors	9.16 (2.27)	8.44 (2.44)	9.46 (2.13)
Design Memory Hits (delay)	9.72 (1.68)	8.77 (1.92)	9.66 (1.80)
Design Memory Correct Distractors (delay)	8.44 (2.49)	7.85 (2.32)	8.59 (2.32)
XO Total Correct (memory)	8.01 (2.34)	7.37 (2.61)	8.71 (2.25)
XO Total Correct (interference)	109.15 (8.64)	103.72 (15.4)	113.16 (9.29)
XO Average Correct Reaction Time (interference)	0.53 (0.07)	0.59 (0.21)	0.50 (0.08)
XO Total Incorrect (interference)	6.89 (4.64)	7.82 (7.30)	6.33 (5.42)
XO Average Incorrect Reaction Time (interference)	0.45 (0.20)	0.50 (0.39)	0.41 (0.13)
Symbol Match Total Correct (visible)	26.73 (1.16)	26.71 (0.96)	26.83 (0.54)
Symbol Match Average Correct Reaction Time (visible)	1.64 (0.53)	1.75 (0.57)	1.58 (0.43)
Symbol Match Total Correct (hidden)	6.05 (2.01)	5.74 (2.09)	7.06 (1.84)
Symbol Match Average Correct Reaction Time (hidden)	1.61 (0.59)	1.71 (0.69)	1.67 (0.66)
Color Match Total Correct	8.57 (1.47)	8.57 (1.21)	8.86 (0.58)
Color Match Average Correct Reaction Time	0.79 (0.17)	0.86 (0.21)	0.77 (0.15)
Color Match Total Commissions	0.47 (1.00)	0.81 (1.44)	0.29 (0.74)
Color Match Average Commissions Reaction Time	0.23 (0.38)	0.31 (0.47)	0.15 (0.34)
Three Letters Total Sequence Correct	3.94 (0.98)	3.47 (1.42)	4.40 (0.91)
Three Letters Average Time to First Click	2.40 (0.73)	2.39 (0.68)	2.10 (0.57)
Three Letters Average Counted Correctly	13.94 (4.18)	13.62 (4.57)	16.26 (4.28)

Table S2. Means and Standard Deviations for ImPACT-Generated Composite Scores by Time Point (N = 1553).

**Time Point**	**Verbal Memory**	**Visual Memory**	**Visual Motor Speed**	**Reaction Time**	**Impulse Control**
	Mean (SD)	Mean (SD)	Mean (SD)	Mean (SD)	Mean (SD)
T1	82.31 (9.98)	72.33 (13.03)	34.56 (6.77)	0.62 (0.09)	7.36 (4.93)
T2	76.01 (14.70)	66.17 (14.97)	33.40 (7.99)	0.68 (0.15)	8.63 (8.03)
T3	86.91 (10.38)	75.30 (12.65)	38.54 (6.94)	0.60 (0.09)	6.63 (5.76)

Note. Composite scores within their domain significantly decreased from T1 to T2 and significantly increased from T2 to T3 after $p < .017$ (.05/3 Bonferroni correction). T1 = baseline assessment, T2 = initial administration of ImPACT within 72 hours of suspected concussion, T3 = second administration of ImPACT within 14 days of suspected concussion.

Figure S2. Estimated Node Strength and Expected Influence Over Time.

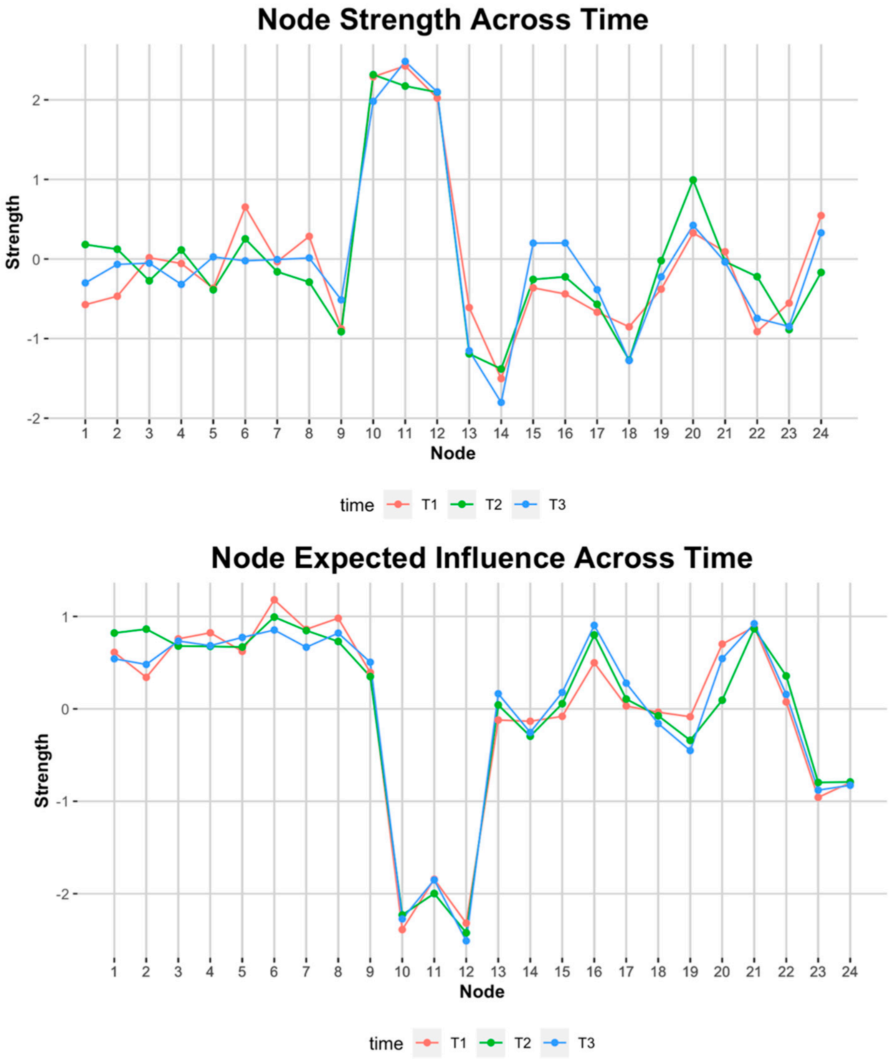

Note. Node strength (top) and expected influence (bottom). Centrality indices are shown as standardized *z*-scores.

Figure S3. Ordered Node Strength Centrality Values at Each Time Point.

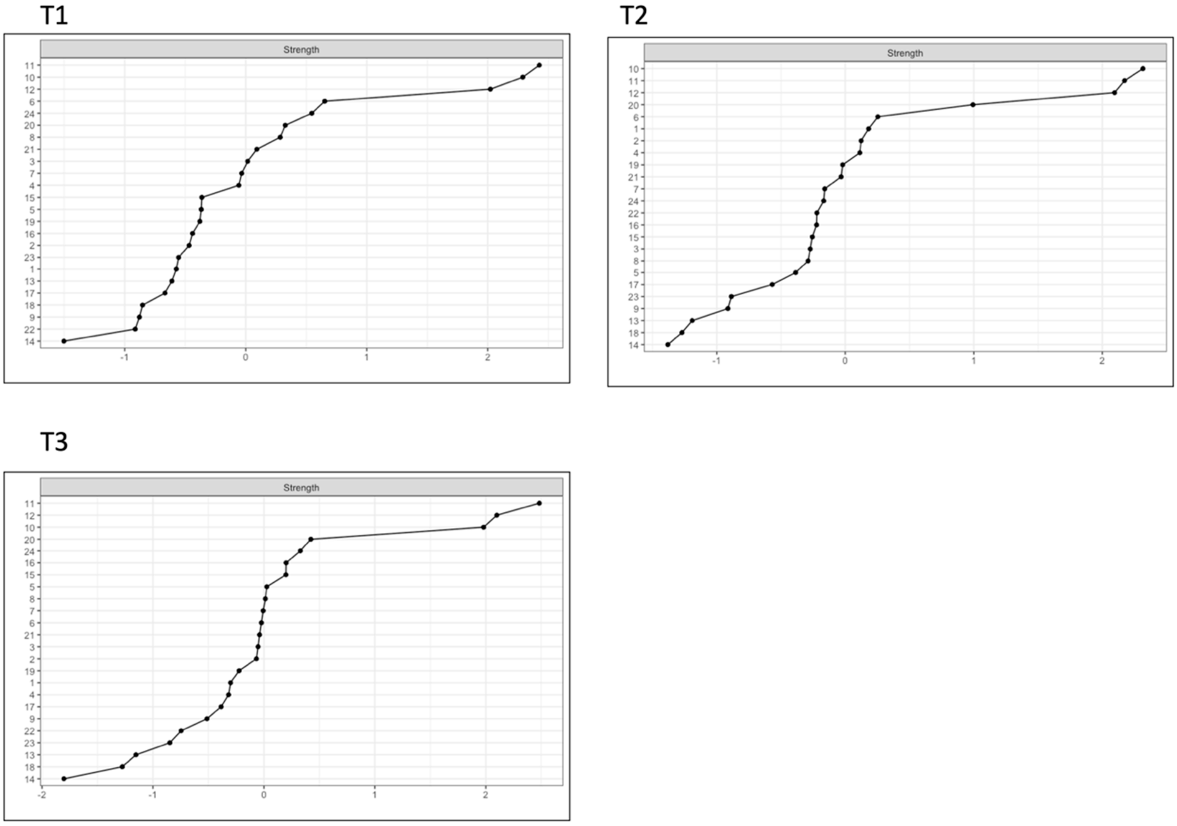

Note. Nodes are presented in order from lowest to highest strength. T1 (top left), T2 (top right), and T3 (bottom left). Centrality indices are shown as standardized *z*-scores.

Figure S4. Ordered Node Expected Influence Values at Each Time Point.

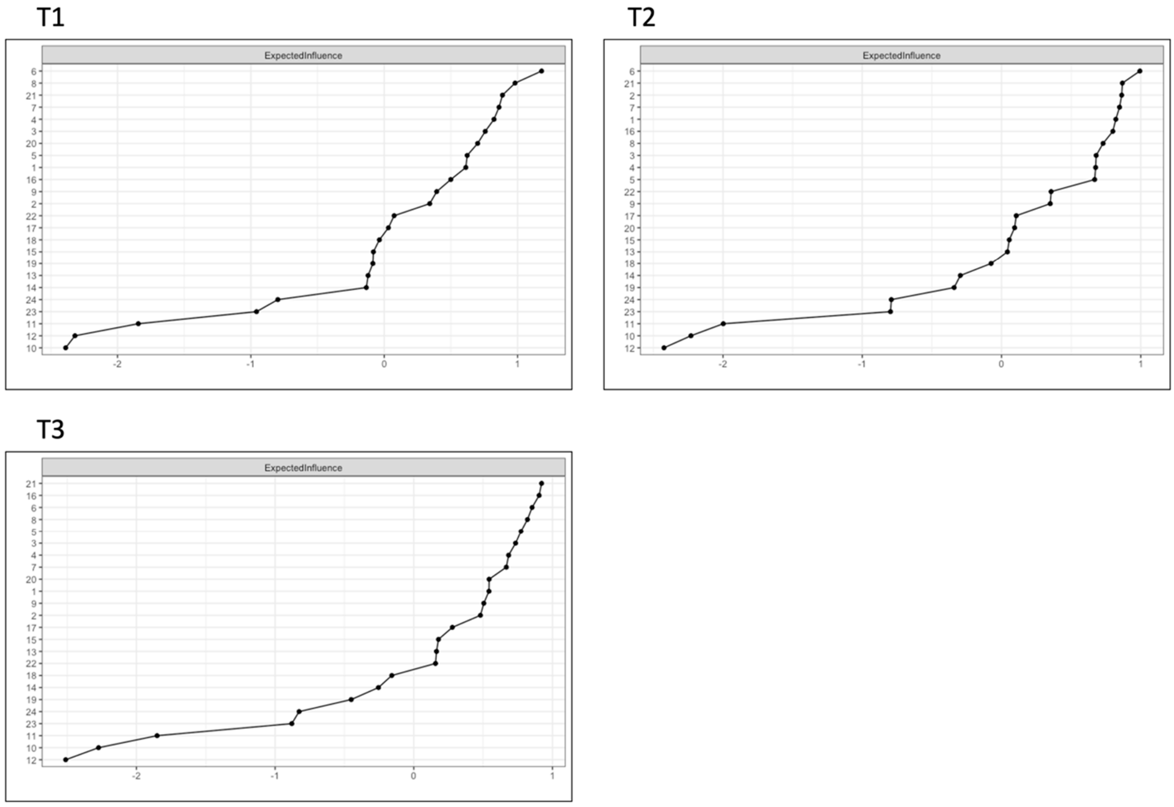

Note. Nodes are presented in order from lowest to highest expected influence. T1 (top left), T2 (top right), and T3 (bottom left). Centrality indices are shown as standardized *z*-scores.

Figure S5. Average Correlations Between Centrality Indices of Networks Sampled with Persons Dropped and Original Sample.

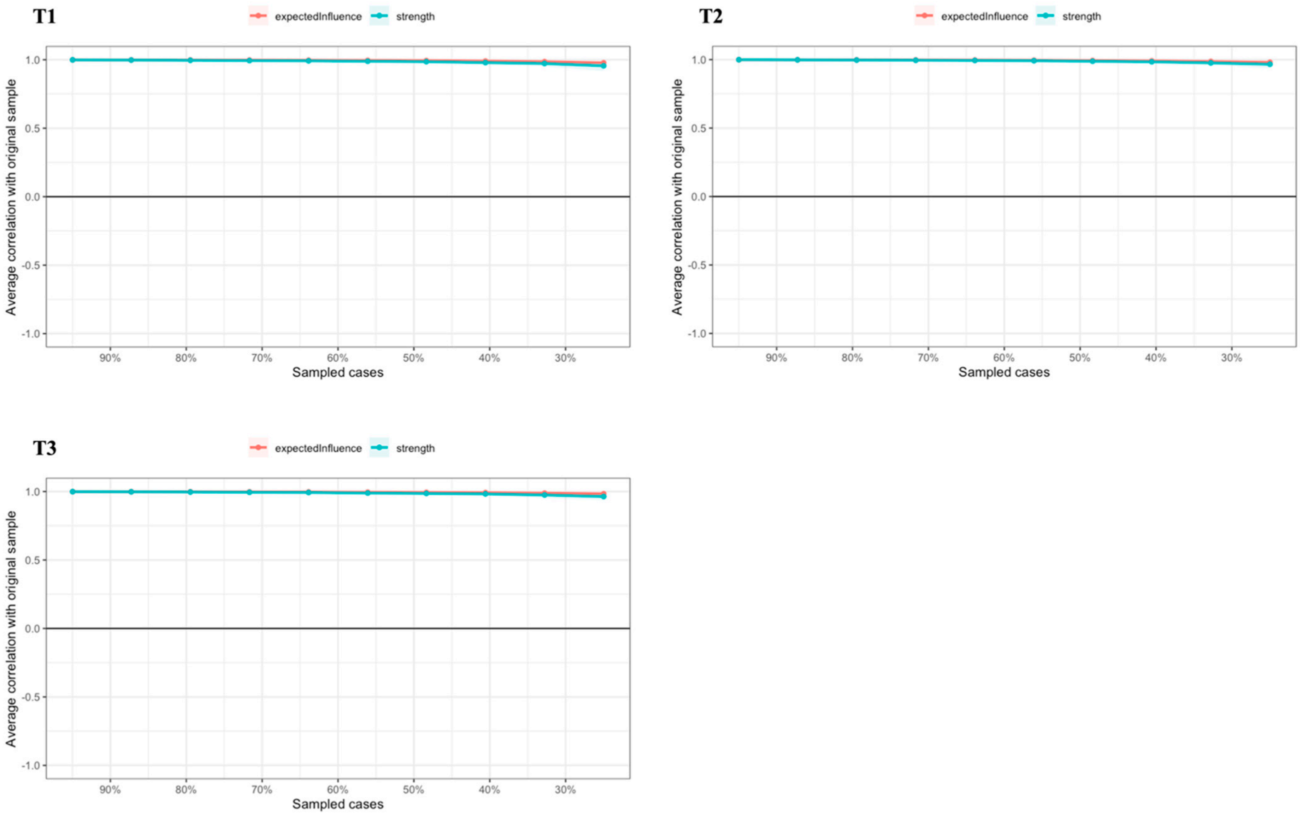

Note. T1 (top left), T2 (top right), and T3 (bottom left). Strength results are shown in blue and expected influence results are shown in pink.

Figure S6. Bootstrapped Confidence Intervals of Estimated Edge-Weights for Each Network.

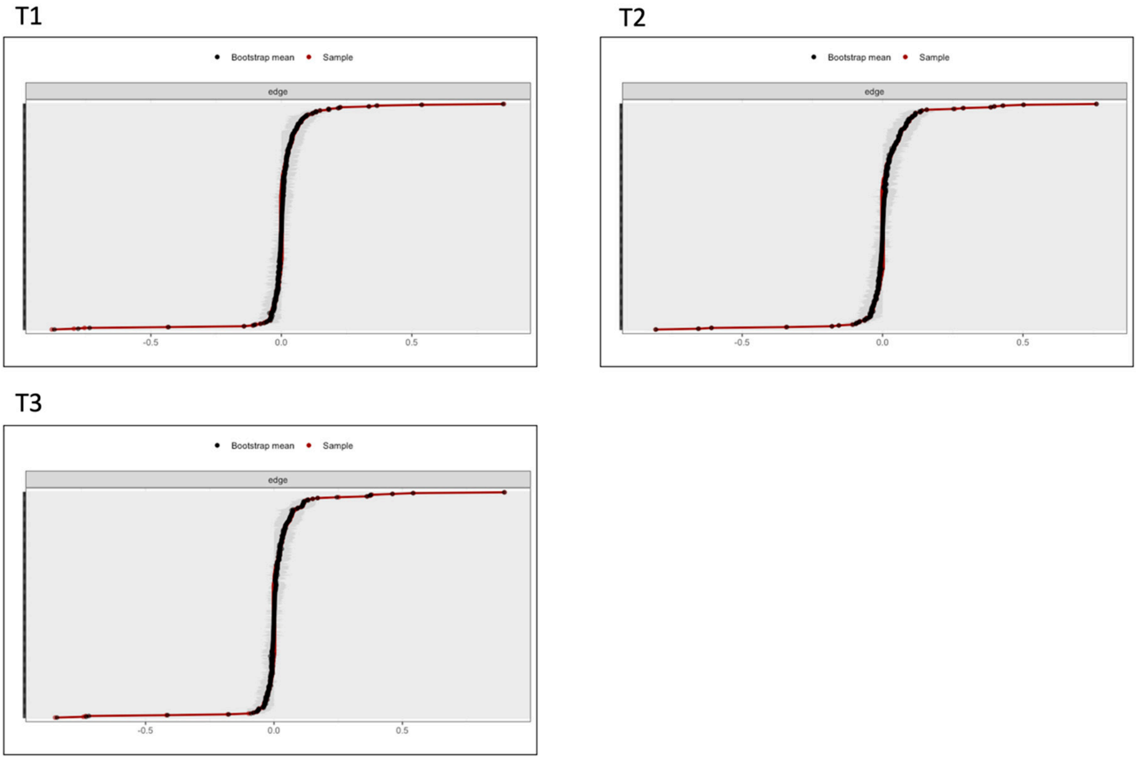

Note. T1 (top left), T2 (top right), and T3 (bottom left). Red lines represents sample values. Gray lines represent bootstrapped confidence intervals. Each horizontal line represents one edge from the network. Edges are ordered from lowest to highest edge-weight.

Figure S7. Bootstrapped Difference Tests Between Edge-Weights That Were Non-Zero in the Estimated Networks.

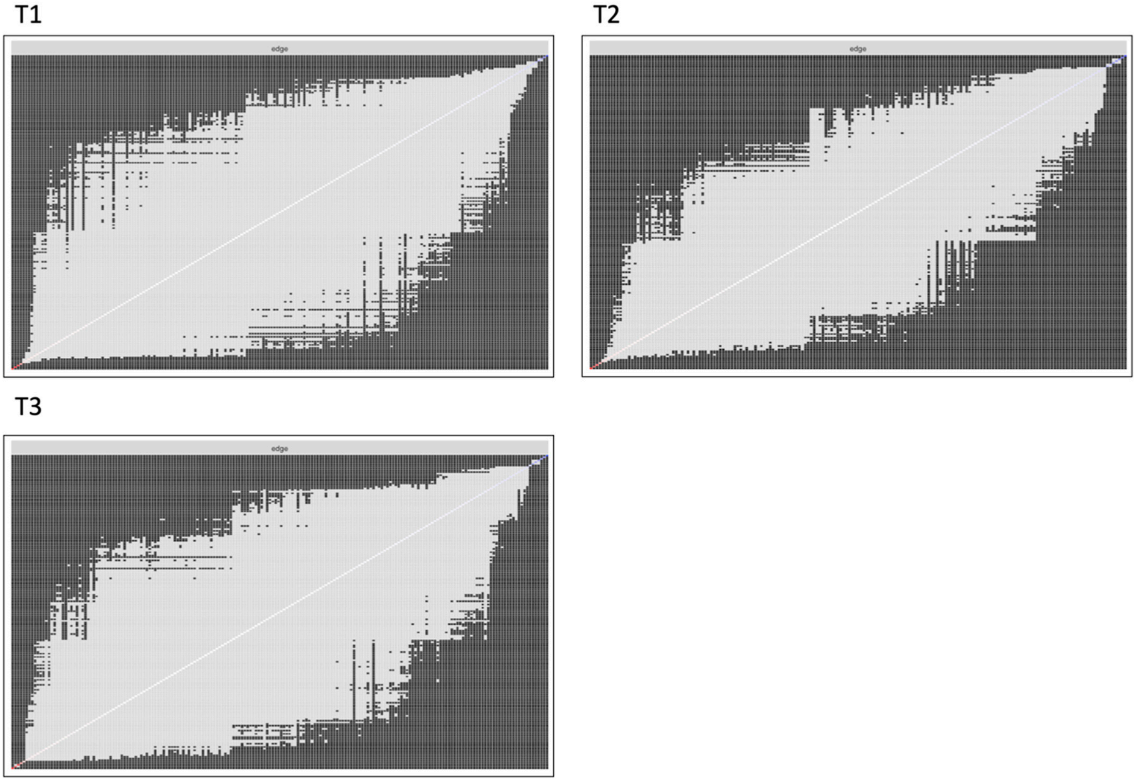

Note. T1 (top left), T2 (top right), and T3 (bottom left). Each unique node pair is represented on both the X and Y axes. Axis labels were removed for clarity. Gray boxes represent edges that do not significantly differ from one-another. Black boxes represent edges that do significantly differ from one another. Colored boxes correspond to the color of the edge in Figure 1.

Figure S8. Bootstrapped Difference Tests Between Node Strength in the Estimated Networks.

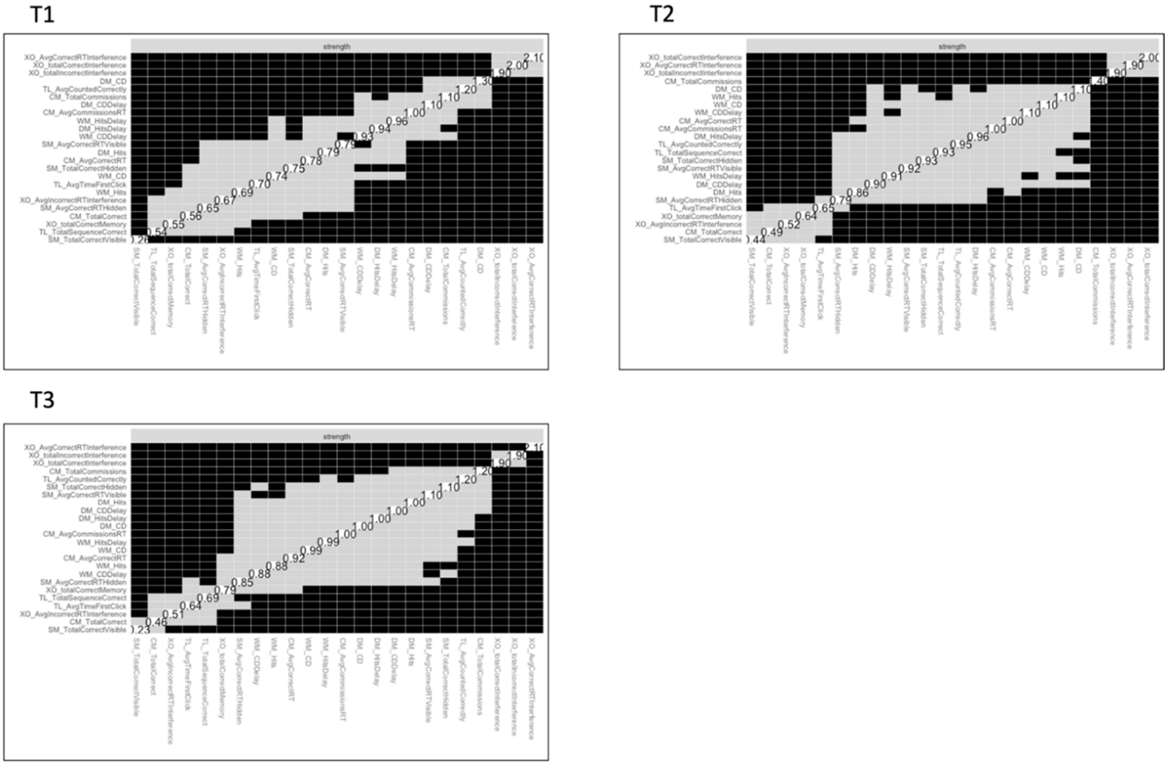

Note. T1 (top left), T2 (top right), and T3 (bottom left). Each node is represented once on the X axes and once on the Y axes. Gray boxes represent nodes that do not significantly differ from one-another. Black boxes represent nodes that do significantly differ from one another. White boxes show the value of node strength.

Figure S9. Bootstrapped Difference Tests Between Expected Influence in the Estimated Networks.

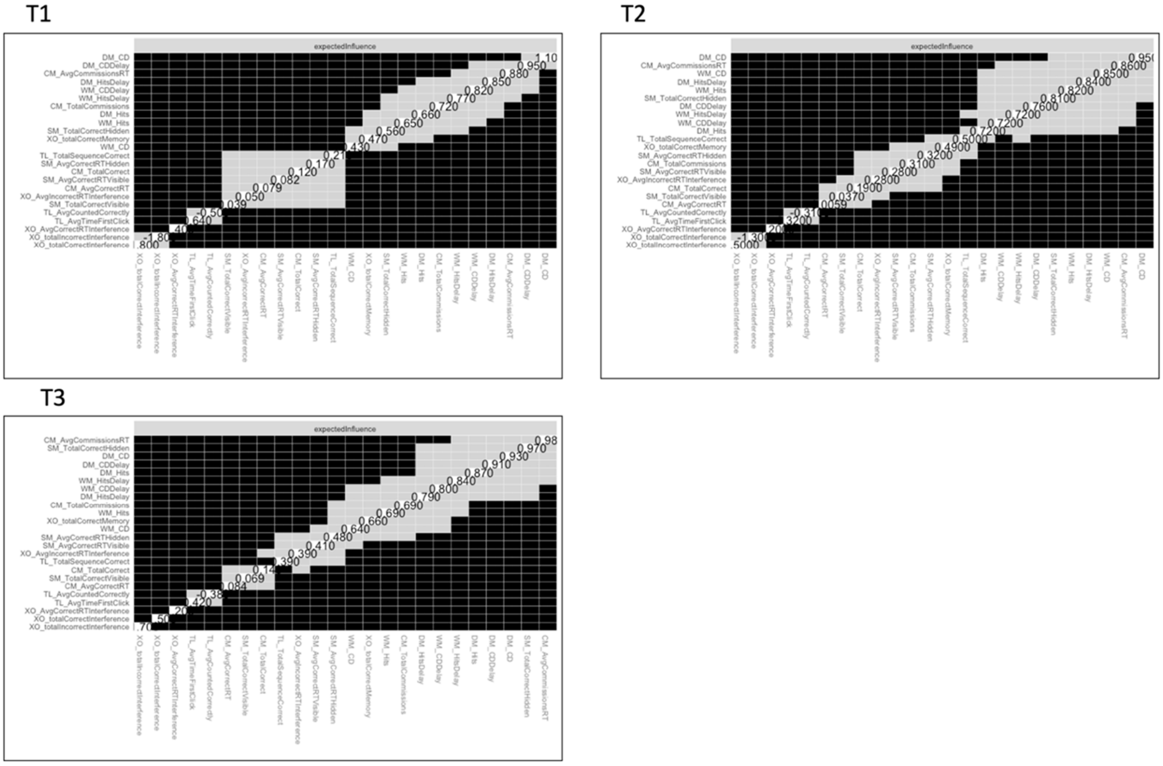

Note. T1 (top left), T2 (top right), and T3 (bottom left). Each node is represented once on the X axes and once on the Y axes. Gray boxes represent nodes that do not significantly differ from one-another. Black boxes represent nodes that do significantly differ from one another. White boxes show the value of node expected influence.
